# Endovascular Embolization for Traumatic Facial Artery Pseudoaneurysm: A Case Report

**DOI:** 10.7759/cureus.72349

**Published:** 2024-10-25

**Authors:** Meghdad Ghasemi Gorji, Fardin Karbakhsh Ravari, Ali Rafiei

**Affiliations:** 1 Department of Vascular Surgery, Shiraz University of Medical Sciences, Shiraz, IRN

**Keywords:** embolization, facial artery, interventional radiology, pseudoaneurysm, trauma

## Abstract

This article presents a rare case of a facial artery pseudoaneurysm that developed following blunt trauma to the right buccal region. The patient, a 75-year-old male, sought medical attention four days after sustaining an injury from a stone impact, reporting pain and swelling in the right mandibular area. During the physical examination, a pulsatile mass was identified in the region of the facial artery. Color Doppler ultrasound confirmed the presence of a facial artery pseudoaneurysm, which was further validated by CT angiography. A team of vascular surgeons and interventional radiologists opted for endovascular embolization as the treatment approach. The procedure, performed under local anesthesia using embolic coils, was completed without complications. Follow-up imaging at two weeks and three months post-procedure confirmed successful occlusion of the pseudoaneurysm, with no signs of recurrence. This case illustrates that endovascular embolization is a safe and effective treatment for facial artery pseudoaneurysms, offering a minimally invasive alternative to traditional surgical methods that may carry risks such as scarring and excessive bleeding.

## Introduction

Pseudoaneurysms occur when a breach in the arterial wall leads to blood leakage into surrounding tissue, forming a pulsatile hematoma. While trauma is a common cause, pseudoaneurysms of the facial artery are rare due to their protected position [1]. These injuries often present as painful, pulsatile masses following facial trauma.

Traditionally, facial artery pseudoaneurysms have been managed with surgical ligation, which provides direct control over complex cases and allows for immediate resolution of the pseudoaneurysm. However, surgical treatment carries risks, including bleeding, infection, and longer recovery times. In contrast, modern advances in interventional radiology, such as endovascular embolization, offer a less invasive alternative. This approach, which includes techniques like coil embolization, particle embolization (e.g., polyvinyl alcohol), and glue (N-butyl-2-cyanoacrylate, NBCA), is associated with faster recovery, fewer complications, and minimal tissue disruption. However, it may not be as effective in treating more complex lesions, particularly in cases of large or multilobed aneurysms. Additionally, endovascular embolization has several technical limitations, including difficulty in accessing smaller or tortuous vessels, particularly in cases of small caliber arteries. Furthermore, this approach may be contraindicated in patients with active infections due to the risk of spreading infection along the vascular pathways, and there is also a risk of incomplete embolization or recanalization of the vessel over time [2,3].

This case report highlights a rare instance of a traumatic facial artery pseudoaneurysm successfully treated with endovascular embolization, demonstrating its effectiveness and safety.

## Case presentation

**Figure 1:**
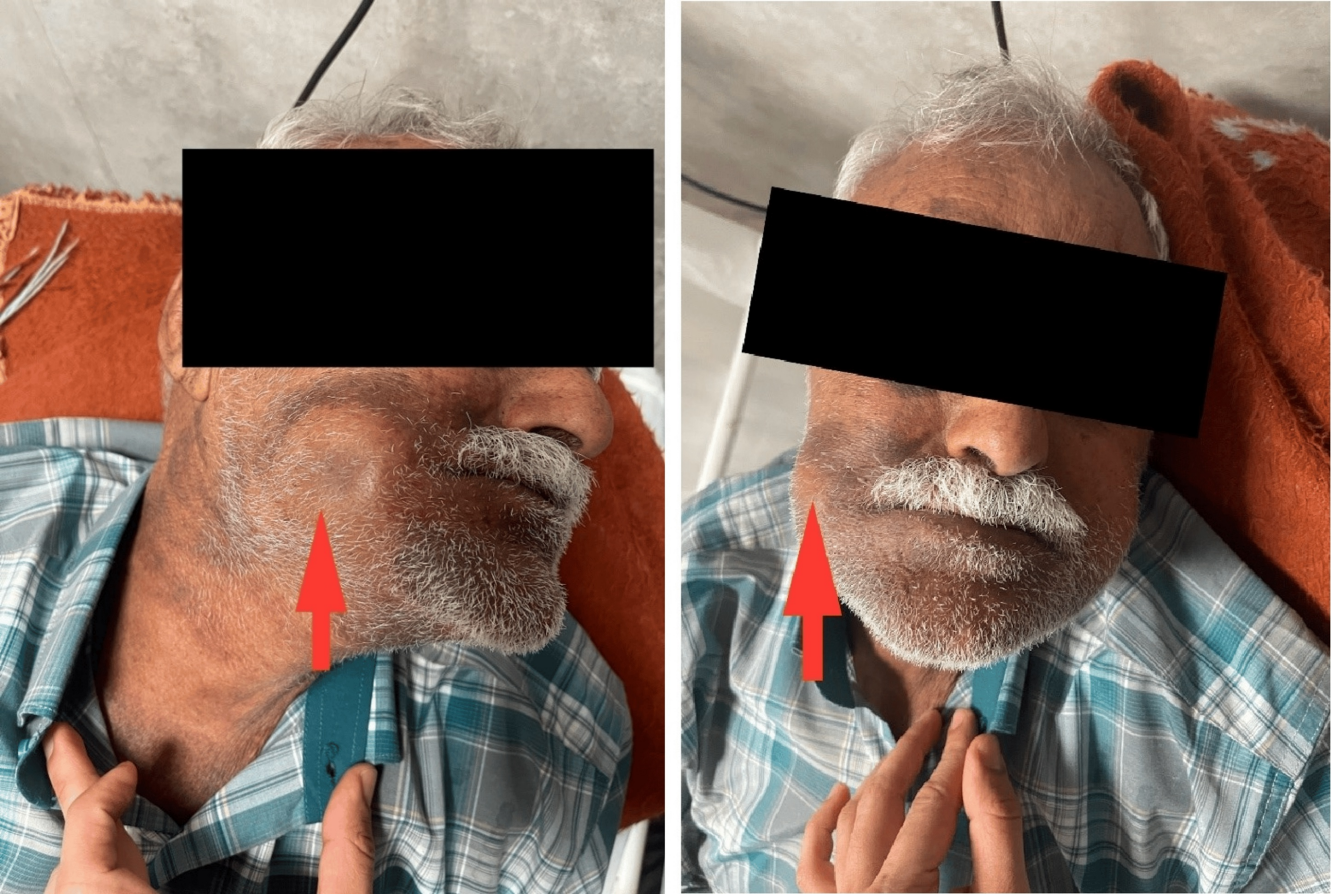
Right-side pulsatile mass in the buccal region

A 75-year-old Iranian male with an unremarkable past medical history presented four days after being struck by a stone thrown by another individual during an altercation. The impact occurred on the right buccal region. Following the injury, he experienced localized pain in the right mandible, which was followed by gradual swelling at the site of impact. The lesion progressively enlarged over the four days but was not associated with any neurological deficits. On the fourth day, after significant swelling had developed, a physical examination revealed a pulsatile mass in the region of the facial artery (Figure 1).

**Figure 2:**
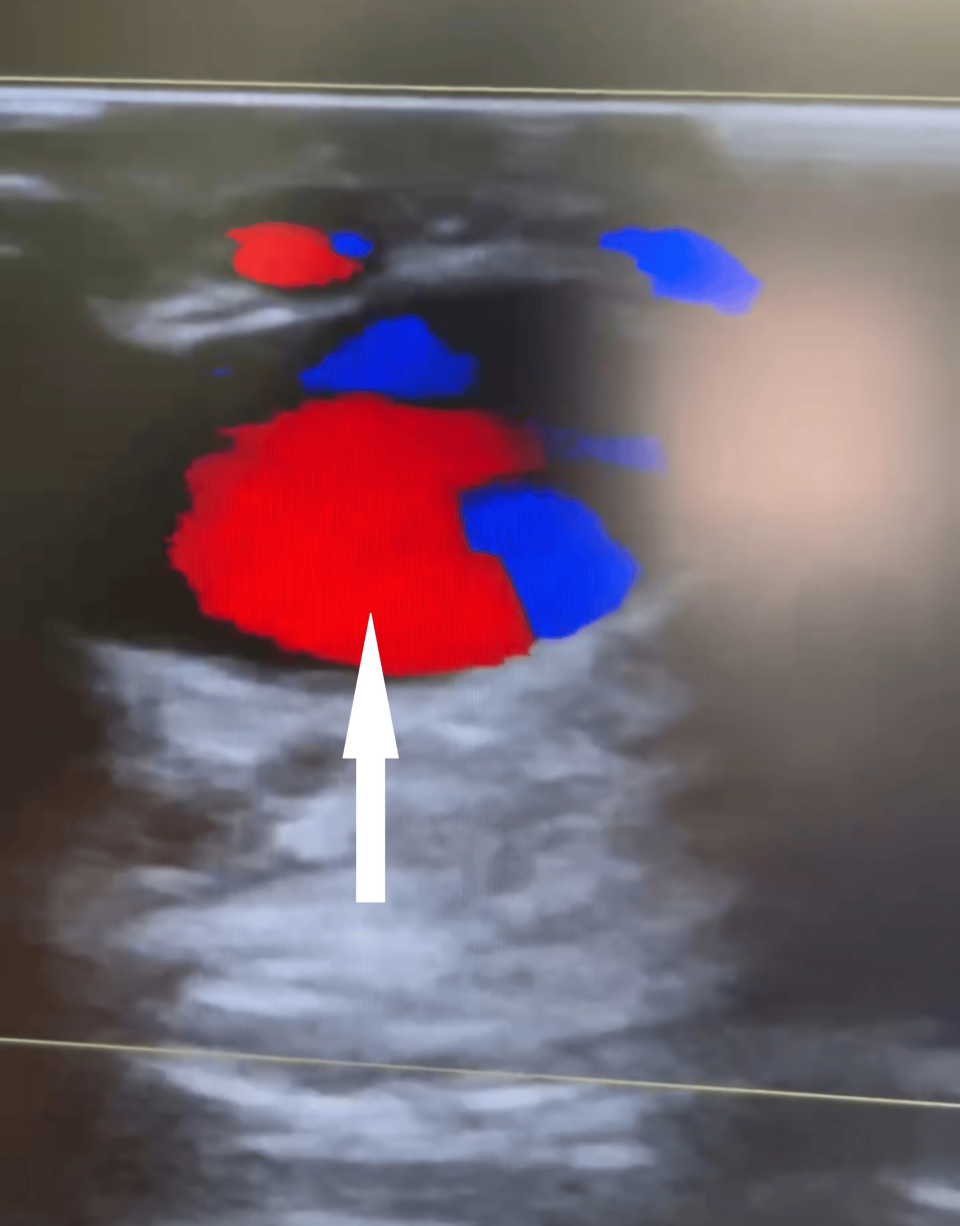
Color Doppler ultrasound demonstrating the presence of a facial artery pseudoaneurysm in the right buccal region, with clear visualization of turbulent blood flow within the lesion

**Figure 3:**
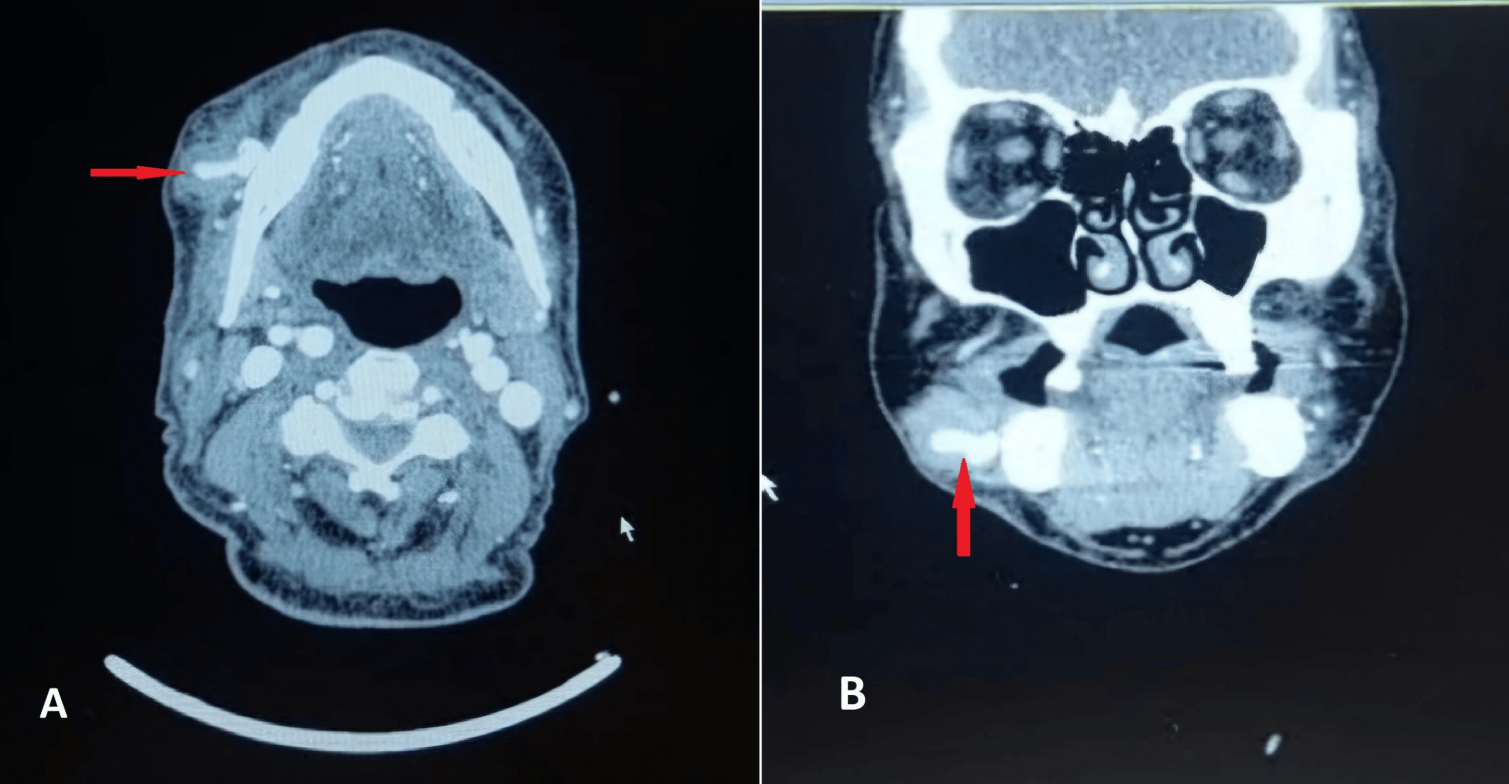
Pseudoaneurysm of the facial artery depicted in CT angiography, shown in axial (A) and coronal (B) planes

A color Doppler ultrasound was performed, revealing a pseudoaneurysm of the facial artery (Figure 2). This finding was subsequently confirmed by CT angiography (Figure 3).

**Figure 4:**
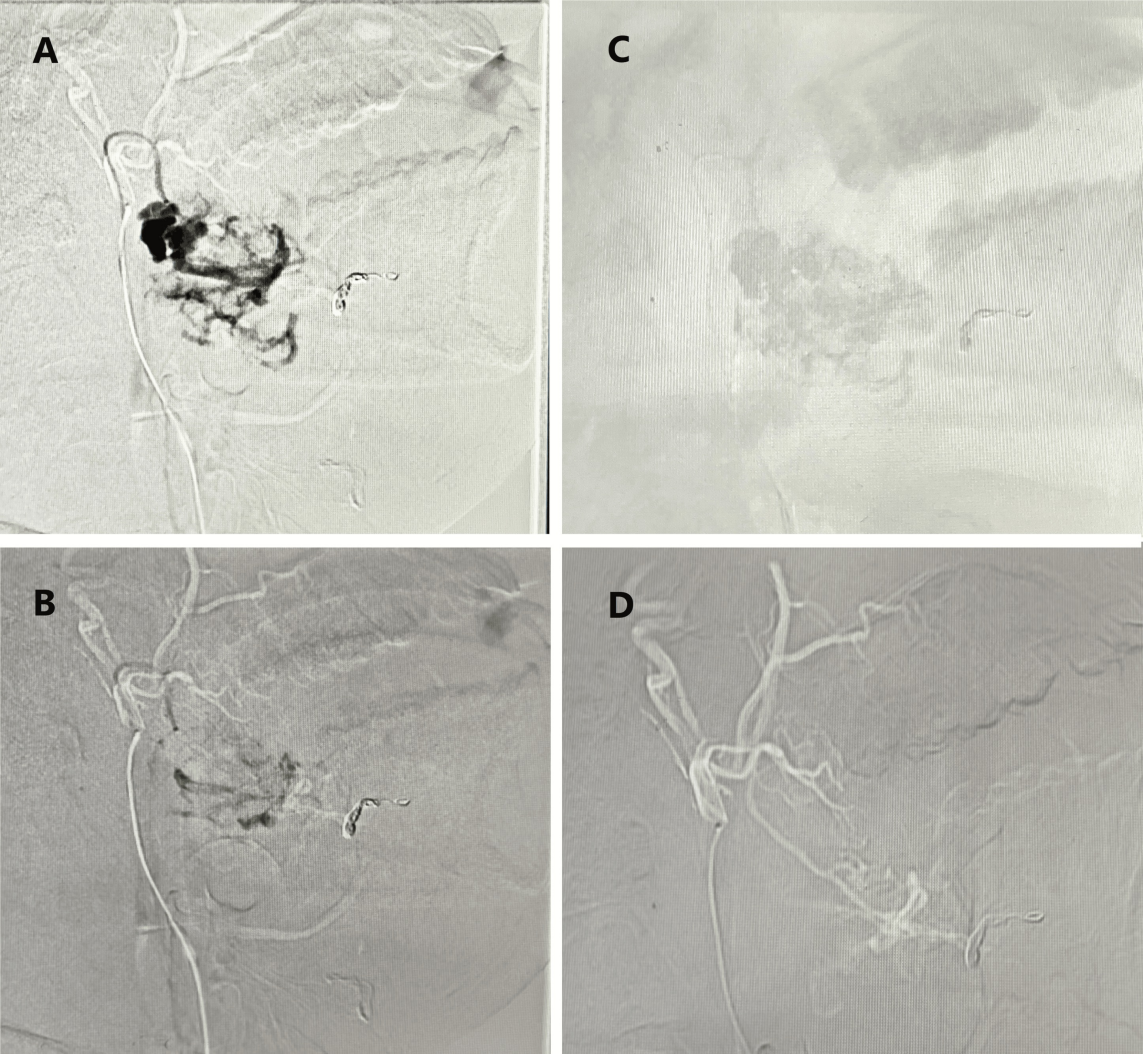
(A) Femoral access to the right external carotid artery using a 7F guiding catheter. (B) Contrast injection confirming the appropriate site, followed by the deployment of NBCA glue and microcoil within the facial artery pseudoaneurysm. (C) Microcoil positioned inside the facial artery pseudoaneurysm. (D) Final stage of endovascular microcoil embolization demonstrating successful occlusion of the pseudoaneurysm NBCA, N-butyl-2-cyanoacrylate

A multidisciplinary team, including vascular surgeons and interventional radiologists, evaluated the case and opted for endovascular embolization as the primary treatment. Femoral access was obtained using a 7F standard guiding sheath and a 7F guiding catheter (Sidekick® Tapered, BD Interventional, Franklin Lakes, New Jersey, USA) to navigate to the right external carotid artery. After confirming the site of extravasation through contrast injection, the artery was initially embolized using NBCA glue to block blood flow. Subsequently, a microcoil (Tornado® Embolization Microcoil, Cook Medical, Bloomington, Indiana, United States, 2-5 mm diameter, platinum with nylon fiber) was deployed into the pseudoaneurysm to ensure complete occlusion (Figure 4). The procedure was successfully performed under local anesthesia, and no immediate complications were observed. Post-procedure monitoring revealed no signs of bleeding, nerve dysfunction, or ischemia. A follow-up Doppler ultrasound performed two weeks later confirmed successful occlusion and resolution of the aneurysm.

Given the success of the embolization, surgical intervention was not required. The patient received guidance on post-procedure care, wound management, and sun protection. At the three-month follow-up, CT angiography confirmed the complete resolution of the pseudoaneurysm, with no evidence of recurrence. The minimally invasive embolization approach proved effective, allowing the patient to recover without complications or the need for further interventions.

## Discussion

Pseudoaneurysms of external carotid artery branches are rare and typically result from trauma. Facial artery pseudoaneurysms commonly arise from the distal vessel, which crosses the mandible and is vulnerable to injury [2]. These lesions typically present as a pulsatile, painful swelling in the affected region. Diagnostic imaging modalities, including ultrasound, CT, and angiography, are essential for confirmation [4].

Pseudoaneurysms can be treated by ligating the affected blood vessel, which can then be surgically removed with minimal risk to tissue perfusion, particularly in the case of the facial artery [5]. However, surgery has limitations, such as scarring and excessive bleeding. Advances in interventional radiology, particularly endovascular embolization, offer a less invasive alternative with excellent outcomes. This method is particularly useful for difficult-to-access aneurysms, reducing the risks of delayed bleeding and revascularization. While generally safe, complications like neurological damage or tissue necrosis from embolization material have been reported [4,6].

Some studies highlight the successful use of microcoils and liquid embolic agents like Onyx in treating vascular lesions, offering durable and satisfactory results while minimizing the risk of microvascular complications. In our case, embolic microcoils and NBCA ensured complete occlusion of the pseudoaneurysm without immediate complications [7,8]. However, as Hetts et al. (2012) noted, rare complications, such as delayed transcutaneous extrusion of embolic microcoils, can occur, emphasizing the importance of careful post-treatment monitoring [9]. Similar to the findings of Pukenas et al. (2012), our follow-up two weeks after the embolization confirmed the successful obliteration of the pseudoaneurysm [8]. These outcomes demonstrate the growing reliance on endovascular embolization as a viable alternative to surgical intervention, particularly in managing rare facial artery pseudoaneurysms.

## Conclusions

Facial artery pseudoaneurysms are uncommon but can arise after an injury, posing challenges due to their location and potential complications. This case illustrates that endovascular embolization is a minimally invasive and highly effective treatment option, offering advantages over traditional surgery. Embolization represents a significant advancement in managing facial vascular injuries, allowing for precise closure with minimal risk of scarring, bleeding, or nerve damage. This approach ensures swift recovery and underscores the increasing role of interventional radiology in treating complex vascular conditions, ultimately leading to safer, patient-focused care.
